# Correlation of Obesity With Elevated Blood Pressure Among Racial/Ethnic Minority Children in Two Los Angeles Middle Schools

**Published:** 2008-03-15

**Authors:** Antronette K Yancey, William J McCarthy, Judith M Siegel, Weng Kee Wong, Andriette Ward, Joanne Leslie, Eloisa Gonzalez

**Affiliations:** Department of Health Services, UCLA School of Public Health. Dr Yancey also is affiliated with the Division of Cancer Prevention and Control Research, UCLA Jonsson Comprehensive Cancer Center, and the UCLA Center for Health Policy Research, Los Angeles, California; Department of Health Services, UCLA School of Public Health, Los Angeles, California. Dr McCarthy also is affiliated with the Division of Cancer Prevention and Control Research, UCLA Jonsson Comprehensive Cancer, and the Department of Psychology, UCLA College of Letters and Sciences Center and School of Public Health, Los Angeles, California; Department of Community Health Sciences, UCLA School of Public Health, Los Angeles, California; Department of Biostatistics, UCLA School of Public Health, Los Angeles, California; Andriette Ward, Children’s Hospital Los Angeles, Los Angeles, California; Children’s Hospital Los Angeles, Los Angeles, California; Visiting Scholar, Women’s Studies Program, UCLA College of Letters and Sciences, Los Angeles, California; Department of Health Services, Public Health Branch, Los Angeles County, California

## Abstract

**Introduction:**

To identify anthropometric and fitness correlates of elevated blood pressure, serum cholesterol, and glycated hemoglobin, we examined anthropometric and physiologic biomarkers among racial/ethnic minority children aged 11 to 13 years in two urban Los Angeles middle schools. We explored the potential for using obesity or fitness level as screening variables for cardiovascular disease risk factors in these students.

**Methods:**

During regularly scheduled physical education classes, we collected data on demographic characteristics, height, weight, blood pressure, nonfasting total serum cholesterol, glycated hemoglobin, time to run/walk 1 mile, and a range of self-reported behaviors. A total of 199 sixth-graders (121 Latinos, 78 African Americans) participated in the study.

**Results:**

Bivariate analyses indicated that 48.6% of sixth-graders were of desirable weight, 17.5% were overweight, 29.9% were at risk for overweight, and 4.0% were underweight. Higher weight was associated with higher levels of serum cholesterol, systolic blood pressure, and diastolic blood pressure (*P* values for all associations <.02) but not with glycated hemoglobin. Multivariate analyses maintained the findings with regard to blood pressure but not serum cholesterol.

**Conclusion:**

Overweight status could be a screening variable for identifying youth at risk for high blood pressure. Obesity prevention and intervention programs and policies need to target low-income racial/ethnic minority children. Assessment of hypertension status also seems warranted in low-income racial/ethnic minority sixth-graders, as does early intervention for children at high risk.

## Introduction

Obesity among U.S. adolescents has reached epidemic proportions, particularly for African Americans and Latinos (1). In some socioeconomically disadvantaged communities, rates of overweight approach 50% among elementary school–aged children (2). Obesity-related chronic diseases previously found in adults increasingly affect youth. For example, nearly one-third of type 2 diabetes mellitus has onset during in the second decade of life, primarily in youth of color, usually in association with obesity (3,4). A 32-year follow-up study of Norwegian teens yielded death rates 80% to 100% higher for those with body mass index (BMI) at or above the 95th percentile at baseline (5). In the Bogalusa Heart Study, overweight was highly predictive of cardiovascular disease (CVD) risk factor clustering in children as young as 7 years (6) and thus had potential as an effective screening variable for CVD risk factors.

Recognized CVD risk factors in children include high blood pressure (BP), elevated serum cholesterol, and elevated glycated hemoglobin (HbA1c) (7). Early intervention during childhood can reduce target organ damage associated with uncontrolled hypertension and other risk factors for CVD (8). However, without government-funded pediatric screening for CVD risk factors, most elevated markers for risk go undetected. Because 30% of overweight children have elevated BP, body composition assessment could identify children at high risk for CVD. Pediatric standards of care include assessment of body composition; such assessment — for example, the Fitnessgram requirement in California (9) — increasingly is required of schools. The potential for using school-based body composition assessment to identify middle-school children at risk for hypertension is particularly important for low-income racial/ethnic minority children living in low-income urban areas who have poor access to medical care.

Despite growing recognition of the pervasiveness of obesity in youth of color, few intervention studies have demonstrated efficacy in these populations (10). Hinkle, documenting the paucity of this research, described efforts to address the gap through pilot projects in community-based organizations (11). Some findings show promise in regard to diet (i.e., decreasing dietary fat, increasing fruit and vegetable consumption), physical activity (i.e., increasing physical activity levels, decreasing sedentary behavior), and body composition (i.e., decreasing body weight, slowing weight gain, reducing body-fat percentage) (12-16), but best practices are difficult to identify because of limitations imposed by small or unrepresentative samples, poorly controlled studies, and reliance on self-reported outcomes.

We present baseline data from the University of California, Los Angeles (UCLA), Community Steps to Minority Youth Fitness (CSMYF) project, a physical education (PE) replacement intervention funded jointly by the National Heart, Lung, and Blood Institute; National Institute of Diabetes and Digestive and Kidney Diseases; National Cancer Institute; and National Institute of Child Health and Human Development to address the worsening obesity epidemic among socioeconomically disadvantaged racial/ethnic minorities. CSMYF involved a partnership of the Los Angeles Unified School District, the Division of Chronic Disease Prevention and Health Promotion in the Los Angeles County Department of Public Health, and researchers from the UCLA School of Public Health. Here we report only the analysis of the baseline data to identify emerging correlates of three CVD risk factors: elevated BP, elevated serum cholesterol, and elevated HbA1c among low-income racial/ethnic minority sixth-graders who live in an urban area.

## Methods

### Participants

We collected baseline data during the 1999–2000 school year from sixth-graders and follow-up data from seventh-graders during both the 1999–2000 and 2000–2001 school years in two middle schools (the middle schools contained grades 6–8; students aged 11–14 years) in the Crenshaw district of Los Angeles. These schools have a primarily African American and Latino enrollment. The schools were matched for number of students and socioeconomic characteristics.

Using a coin toss, we randomly assigned intervention or control status to the schools. Participants were recruited after designation of each school as intervention or control so PE staff at the control school would know the usual practices would prevail at their school. Students were transferred from regular PE classes to CSMYF classes only at the intervention school. In both schools, a presentation to students during regular PE classes using audiovisual exhibits, testimonials, and incentives for participation informed students about the opportunity to participate in the study. All students enrolled in the sixth grade were potentially eligible to participate in the study, but only students at the intervention school were permitted to enroll in the CSMYF PE-replacement program. Because the students were recruited from their PE classes and were fully capable of participating in those classes, none met the exclusion criteria established by the research team (i.e., a serious medical condition precluding participation in common forms of aerobic exercise, metabolic disorders preventing free choice of foods, or a serious learning disability). UCLA research staff distributed information letters (in English and Spanish) to interested students, with instructions to return the signed consent forms promptly. Permission to participate was required both from students' regular PE instructor and from their parent or legal guardian. The UCLA Human Subjects Protection Committee approved the study protocol.

All eligible sixth-graders at the two participating middle schools who wanted to participate in the study were enrolled (132 students at the intervention school and 98 at the control school). For this report, we analyzed only students who self-identified as exclusively Latino (n = 121) or exclusively African American (n = 78).

### Measures

**Race/Ethnicity**

Students self-reported their race/ethnicity as Latino, African American, Asian American/Pacific Islander, white, American Indian, or "biracial (please specify)." A follow-up open-ended question asked students which choice they preferred. Although "Latino" was not further broken down, information collected as part of the California Healthy Kids Survey (see www.wested.org/hks for more information) showed the Latino distribution in the Los Angeles Unified School District is predominantly Mexican American (63%), with 19% having origins in Central America.

**Anthropometric data**

We assessed the height and weight of students at the two schools at approximately the same time of year and same time of day. Using each school's medical-quality balance beam stadiometer, we measured height and weight with the student standing erect and facing the examiner. Body weight in PE clothes was rounded to the nearest quarter pound and height to the nearest quarter inch; we then converted height and weight to metric measurements to calculate BMI in kilograms per meter squared. We calculated students' age- and sex-adjusted BMI percentiles using the revised growth chart data from the Centers for Disease Control and Prevention (17). We classified students as of desirable weight (5th percentile to less than the 85th percentile), at risk for overweight (85th percentile to less than the 95th percentile), overweight (95th percentile or greater), or underweight (less than the 5th percentile) in accordance with the recommendations of the Expert Committee Recommendations for Obesity Evaluation and Treatment (18,19).

**Blood glucose and serum cholesterol**

An experienced pediatric phlebotomist collected a 5-cc venous blood sample from each participant and placed the samples briefly in coolers while collecting blood samples from other students participating in the study. Immediately after the class in which the blood was collected, the samples were transported to the hospital-based clinical laboratory to determine HbA1c and serum cholesterol levels. HbA1c — a marker for long-term glucose control that reflects average daily serum glucose level for the previous 60 to 90 days — was recorded for each student as a percentage rounded to the nearest one-tenth of a percent. The HbA1c test is highly reproducible and minimally affected by bias (20). Because HbA1c values indicating elevated risk in children have yet to be established, our analyses included HbA1c as a continuous variable. Nonfasting total serum cholesterol was recorded for each student in milligrams per deciliter, rounded to the nearest whole number. Following American Academy of Pediatrics criteria (21), we set the cut point for elevated pediatric serum cholesterol at 200 mg/dL.

**Blood pressure**

A trained member of the research team measured BP with the student seated facing the examiner and the arm extended and resting on a table at the level of the heart. The BP cuff allowed the bladder of the cuff to encircle at least two-thirds of the student's upper arm. As suggested by updated national guidelines (22), the fifth Korotkoff sound was used to define diastolic BP. We determined BP-related height- and sex-specific pediatric risk factor cut points using the most current criteria of the National High Blood Pressure Education Program (8). If systolic and diastolic readings fell into different categories, we assigned students to the category conferring greater risk.

**Timed 1-mile run/walk performance**

Students were instructed to complete the 1-mile run/walk as quickly as possible but not to overexert themselves. Researchers at both schools monitored and recorded each student's time to complete four laps around the school's baseball field, equivalent to 1 mile. Times were assessed in minutes and seconds and converted to minutes and fractions thereof. Time to complete 1 mile is an accepted marker for fitness for healthy adolescents and is a recommended measure of fitness in the Fitnessgram (23). The State of California Department of Education has posted recommended time limits for completing the mile, specific for age groups and for girls and boys (24). Recommended times to complete 1 mile were 9 to 12 minutes for girls aged 11 and 12 years, 8.5 to 11 minutes for 11-year-old boys, and 8 to 10.5 minutes for 12-year-old boys.

### Questionnaire

A self-administered survey, offered in English only, asked about physical activity, nutrition (knowledge and intake), health, and psychosocial status. Bilingual study personnel were available to help students complete the survey. Self-reported diet and physical activity-related behaviors by middle school students tend to be reliable, as reflected in κ statistics that exceeded 60% when responses were compared with responses to identical surveys administered 14 days apart (25).

### Statistical analyses

We first compared descriptive statistics between the intervention and control schools, proceeded with bivariate associations with the three CVD markers, and concluded with multivariate, conditional regression of the three CVD markers onto anthropometric and self-reported health behavior measures. Covariates included age, sex, and race/ethnicity.

## Results

Despite socioeconomic comparability, the two schools differed on several baseline measures (Table 1). Of the 1627 students enrolled at the intervention school, 1027 (63%) were African American, 590 (36%) were Latino, and 10 (1%) were from other racial/ethnic groups. At the control school, of 2108 students enrolled, 630 (30%) were African American, 1458 (69%) were Latino, and 20 (1%) were from other racial/ethnic groups (data not shown). Students at the intervention school had higher diastolic BP values (*t*_184_ = 3.6; *P* = .001) and higher HbA1c scores (*t*_168_ = 2.4; *P* = .005) than students at the control school.

Of the 199 sixth-graders in our study, 47.4% were overweight or at risk for overweight (Table 2). Boys and girls differed only slightly on any of the measured variables (Table 3). African American students had higher diastolic BP, after correction for differences in age and height (*t*_168_ = −2.04; *P* = .04); Latinos had higher average BMI percentile (*t*_175_ = 2.60; *P* = .01).

When we controlled for student age, measures were similar between the two schools, except for slower mile run/walk times at the intervention school (*t*_152_ = 6.96: *P* < .001) and higher diastolic BP adjusted for height and age at the intervention school (*t*_176_ = –2.36; *P* = .02).

The percentage of sixth-graders at risk for overweight ranged from 15.2% among African American boys to 41.5% among Latino boys (Table 2). Cross-tabulations of sex by weight status within racial/ethnic groups showed no sex difference among African Americans or among Latinos. Weight did not differ significantly by sex or by race/ethnicity.

Similarly, neither systolic BP-defined hypertension nor diastolic BP-defined hypertension differed significantly by student sex or race/ethnicity (Table 4).

We compared the means for cholesterol, systolic and diastolic BP, and HbA1c as a function of BMI-for-age risk status (desirable weight, at risk for overweight, overweight, and underweight). One-way unconditional analysis of variance indicated a significant association between BMI-for-age and serum cholesterol (*F*_3,155_ = 4.92; *P* = .003), systolic BP (*F*_3,166_ = 22.43; *P* < .001), diastolic BP (*F*_3,166_ = 10.35; *P* < .001), and 1-mile run/walk time (*F*_3,143_ = 7.29; *P* < .001). We found no relation between weight and HbA1c level. Serum cholesterol differed significantly between students of desirable weight and overweight; serum cholesterol in students at risk for overweight did not differ significantly from that of underweight students (Table 4 and Table 5). For systolic BP, students of desirable weight and at risk for overweight did not differ from one another, but both differed significantly from overweight students. Overall, the average CVD risk factor profile for overweight students was a nonfasting total cholesterol of 189 mg/dL, BP values of 121/78 mm Hg, and a mean HbA1c value of 4.7%.

To determine the impact of overweight on BP, controlling for other variables, we regressed systolic and diastolic BP on sex, racial/ethnic group, age, time to complete a 1-mile run/walk, and BMI-for-age risk status. Time to complete the mile, ranging from 7 minutes to 20 minutes 16 seconds, was a crude measure of physical fitness. We did not compute regression analyses for HbA1c because the bivariate analyses showed no association between BMI-for-age and HbA1c.

Students' times to run/walk 1 mile fell just short of statistical significance in predicting systolic hypertension (odds ratio [OR], 1.15; 95% confidence interval [CI], 0.99–1.33; *P* = .06), but overweight significantly predicted systolic hypertension (OR, 3.42; 95% CI, 1.5–7.8) (Table 6). Overweight students were nearly 3.5 times more likely than students of desirable weight to have systolic BPs in the hyptertensive range. Overweight also contributed to a higher diastolic BP (OR, 8.01; 95% CI, 3.1–20.6). Overweight students were eight times more likely than students of desirable weight to have diastolic BPs in the hyptertensive range. The influence of race/ethnicity on diastolic BP was not significant (OR, 2.56; 95% CI, 1.06–6.17), although Latinos were at greater risk than were African Americans (data not shown). If a student was categorized as hypertensive on the basis of either a systolic or diastolic BP, BMI-for-age was the major predictor (OR, 7.04; *P* < .001).

In contrast, multivariate analysis showed no association between BMI-for-age and elevated serum cholesterol (>200 mg/dL); none of the assessed variables independently predicted elevated serum cholesterol in our student sample.

## Discussion

Our baseline sample of racial/ethnic minority sixth-graders in two inner-city middle schools indicated that nearly one-third (29.9%) were at risk for overweight. Sex or racial/ethnic differences in the prevalence of overweight were minimal. These weight statistics are comparable to the 31% of adolescents observed in central Mexico who were recently assessed as being overweight (26). Even with 30% of middle-school students already overweight, these findings nonetheless may underestimate the extent of obesity in inner-city middle schools because more health-conscious students may be predisposed to volunteer for a school-based nutrition and physical activity program. Because we did not collect data from students who did not participate, we could not gauge whether selection bias affected the generalizability of the data.

Our serum cholesterol and BP findings revealed that overweight, low-income racial/ethnic minority children are at higher risk for CVD than were adolescents in the Bogalusa Heart Study (6). Overweight children aged 11 and 12 years in our study had a mean nonfasting total serum cholesterol of 189 mg/dL (Bogalusa median for 11- to 17-year-olds was 157 mg/dL) and a BP of 112/70 mm Hg (Bogalusa median for children aged 11 to 17 years was 107/66 mm Hg). The mean serum cholesterol value of overweight students was significantly higher than for desirable weight sixth-graders, and both systolic and diastolic BP levels were significantly higher for overweight students than for both desirable weight and at-risk-for-overweight students. Thus, overweight could function as a crude variable for screening youth at risk for high BP but was less useful in identifying those with elevated serum cholesterol. Physical fitness, as reflected by students' 1-mile run/walk time, was not successful in multivariate prediction of any of the CVD risk factors. Of students at risk for systolic hypertension (i.e., prehypertensive, hypertensive, or severely hypertensive), 51.1% also were overweight; for diastolic hypertension, 56.4% also were overweight.

A strength of our study is its focus on Latino and African American middle school children living in a low-income area of Los Angeles. Few previous studies have focused on low-income racial/ethnic minority children in the western United States. A limitation of the findings is the cross-sectional nature of the data, particularly BP. A clinical diagnosis of pediatric hypertension requires several BP assessments distributed over multiple visits (27). The consistent relation between overweight and elevated risk for pediatric hypertension, however, cannot be explained as an artifact of the one-time measurement (25).

Both our logistic regression and the literature support the superior utility of overweight and physical fitness as markers for elevated BP rather than for elevated serum cholesterol (27,28). The measure of physical fitness was imperfect because motivational and social factors are potentially important additional influences on the students' time to complete the 1-mile run/walk. Nonetheless, overweight independently predicted both systolic and diastolic BP and weakly predicted serum cholesterol in these analyses. The odds of an overweight minority youth being classified as hypertensive, either from excessive systolic BP or from excessive diastolic BP, was about eightfold higher than that of their peers of desirable weight. No other nondemographic variable was significantly associated with observed risk for high BP, including physical fitness as measured by the 1-mile run/walk.

Analyses of these baseline data highlight the need for obesity prevention and intervention programs targeted to racial/ethnic minority youth to reduce their current and future risk for hypertension. Bringing overall student nutrition (at school and at home) into compliance with the current Dietary Guidelines for Americans (29) should help reduce risk for hypertension, especially through adherence to dietary sodium standards for African Americans (30), and should contribute to decreased risk for obesity (31). For the nearly 50% of hypertensive children whose hypertension would not have been diagnosed by their overweight status, these results also suggest that direct measurement of BP is indicated. Even though weight status in minority children aged 11 and 12 years could be a marker for other elevated risk factors for CVD (particularly high BP), research is needed to determine whether intervention-mediated changes in weight can reduce risk for pediatric high BP and reduce future risk for high BP in adulthood. The importance of this question is underscored by recent data from the National Health and Nutrition Examination Survey (NHANES) showing a reversal at the end of the last decade in the downward trend of the prevalence of high BP (32). To increase the lifespans of children in this generation, we must identify effective strategies to reverse the high levels of weight-related risk factors for CVD documented here in low-income, racial/ethnic minority children.

## Figures and Tables

**Table 1:** Baseline Characteristics of Sixth-Grade Students (N = 199) in Two Inner-City Middle Schools, Los Angeles, California, 1999–2000 School Year

Characteristic	Intervention School Mean (SD)^a^	Control School Mean (SD)	*P* Value^b^
Age, y	11.6 (0.4)	11.6 (0.5)	.31
African American, n	53	25	.027
Latino, n	63	58	
BMI	21.8 (6.1)	22.5 (6.3)	.44
BMI-for-age percentile	69.4 (30.6)	72.1 (29.5)	.001
Systolic BP, mm Hg	111 (10.9)	113.2 (13.8)	.23
Diastolic BP, mm Hg	72 (14.4)	66 (8.6)	.001
Systolic BP, age- height-, and sex-adjusted percentile	62.7 (28.2)	68.5 (26.8)	.19
Diastolic BP, age- height-, and sex-adjusted percentile	71.1 (24.7)	62.4 (21.4)	.02
Total serum cholesterol (nonfasting), mg/dL	169.8 (30.5)	177.6 (42.3)	.15
HbA1c, %	4.7 (0.4)	4.5 (0.6)	.005

BMI indicates body mass index; BP, blood pressure; HbA1c, glycated hemoglobin.

a Sample sizes vary slightly for individual measures because of missing data.

b All *P* values were obtained from *t *tests except for African American and Latino, for which the *P* value was obtained from a goodness-of-fit chi-square test applied to a 2 x 2 frequency table comparing the frequency of African Americans and Latinos across the two schools.

**Table 2:** Age- and Sex-Adjusted BMI^a^ Weight Status Categories of Sixth-Grade Students (N = 199) by Race/Ethnicity and Sex in Two Inner-City Middle Schools, Los Angeles, California, 1999–2000 School Year

Variable		No. Underweight (%)	No. Desirable Weight(%)	No. at Risk for Overweight(%)	No. Overweight(%)

Race/Ethnicity	Sex				
African American	Female	3 (7.9)	18 (47.4)	12 (31.6)	5 (13.2)
	Male	1 (3.0)	20 (60.6)	5 (15.2)	7 (21.2)
Latino	Female	3 (4.6)	33 (50.8)	19 (29.2)	10 (15.4)
	Male	0	15 (36.6)	17 (41.5)	9 (22.0)
Total^b^		7 (4.0)	86 (48.6)	53 (29.9)	31 (17.5)

BMI indicates body mass index.

a Underweight = less than the 5th percentile; desirable weight = 5th percentile to less than the 85th percentile; at risk for overweight = 85th percentile to less than the 95th percentile; overweight = 95th percentile or greater (defined in accordance with the recommendations of the Expert Committee Recommendations for Obesity Evaluation and Treatment) (19).

b N does not total 199 because of missing data on weight, height, or race/ethnicity.

**Table 3:** Demographic, Anthropometric, and Fitness Variables, by Sex and Race/Ethnicity for Sixth-Grade Students (N = 199) in Two Inner-City Middle Schools, Los Angeles, California, 1999–2000 School Year

Variable	Boys Mean (SD)	Girls Mean (SD)	African American Mean (SD)	Latino Mean (SD)
Age, y	11.6 (0.06)	11.6 (0.04)	NA	NA
Race/Ethnicity, n	NA	NA	64-78^a^	99-121^a^
African American, n	38	40	NA	NA
Latino, n	51	70	NA	NA
BMI, kg/m^2^	22.1 (6.1)	22.0 (6.2)	21.6 (7.4)	22.3 (5.2)
BMI age- and sex-adjusted percentile	71.6 (29.7)	69.7 (30.5)	63.4 (33.2)	75.2^b^ (27.0)
Time to run/walk 1 mile, min	11.3 (2.9)	12.0 (2.8)	11.5 (3.0)	11.8 (2.78
Systolic BP, mm Hg	112 (12.5)	112 (12.0)	111 (10.4)	112 (13.2)
Diastolic BP, mm Hg	69 (11.9)	70 (13.4)	73 (13.8)	68^c^ (11.7)
Systolic BP, age-, height-, and sex-adjusted percentile	63.5 (28.9)	66.0 (27.0)	61.7 (27.9)	67.2 (27.6)
Diastolic BP, age-, height-, and sex-adjusted percentile	67.7 (23.2)	67.6 (24.3)	72.2 (23.2)	64.7 (23.8)
Total serum cholesterol (nonfasting), mg/dL	173.2 (33.1)	172.5 (37.8)	167.6 (42.3)	176.2 (30.6)
HbA1c, %	4.5 (0.5)	4.7 (0.4)	4.7 (0.5)	4.6 (0.5)

NA indicates not applicable; BMI, body mass index; BP, blood pressure; HbA1c, glycated hemoglobin.

a Sample sizes vary slightly for individual measures because of missing data.

b *P* < .05 African American vs Latino.

c *P* < .01 African American vs Latino.

**Table 4:** Systolic and Diastolic Blood Pressure Categories of Sixth-Grade Students (N = 199) by Race/Ethnicity and Sex in Two Inner-City Middle Schools, Los Angeles, California, 1999–2000 School Year

Race/Ethnicity	Sex	Systolic Blood Pressure^a^				Total

		No. Desirable BP(%)	No. Prehypertensive(%)	No. Hypertensive(%)	No. Severely Hypertensive(%)	
African American	Female	30 (79.0)	4 (10.5)	2 (5.3)	2 (5.3)	38
	Male	24 (82.8)	3 (10.3)	1 (3.4)	1 (3.4)	29
Latino	Female	44 (68.8)	8 (12.5)	9 (14.1)	3 (4.7)	64
	Male	27 (69.2)	4 (10.3)	4 (10.3)	4 (10.3)	39
Total		125 (73.5)	19 (11.2)	16 (9.4)	10 (5.9)	170^b^ (100)

**Race/Ethnicity**	**Sex**	**Diastolic Blood Pressure^a^**				**Total**

		**No. Desirable BP(%)**	**No. Prehypertensive(%)**	**No. Hypertensive(%)**	**No. Severely Hypertensive(%)**	

African American	Female	26 (68.4)	3 (7.9)	3 (7.9)	6 (15.8)	38
	Male	21 (72.4)	6 (20.7)	2 (6.9)	0	29
Latino	Female	55 (85.9)	3 (4.7)	2 (3.1)	4 (6.2)	64
	Male	29 (74.4)	7 (18.0)	1 (2.6)	2 (5.1)	39
Total		131 (77.1)	19 (11.2)	8 (4.7)	12 (7.1)	170^b^ (100)

a Desirable blood pressure was <90th percentile for height; prehypertensive was ≥90th percentile but <95th percentile for height; hypertensive was ≥95th percentile but <99th percentile for height; severely hypertensive was ≥99th percentile for height (22).

b N does not total 199 because of missing data on weight, height, or race/ethnicity.

**Table 5:** Mean Anthropometric and Medical Measures of Sixth-Grade Students (N = 199), by Body Mass Index-for-Age, in Two Inner-City Middle Schools, Los Angeles, California, 1999–2000 School Year

**BMI Category^a^**	Serum Cholesterol, mg/dL (SD)^b^	HbA1c, % (SD)	Systolic BP Percentile^b^ (SD)	Diastolic BP Percentile^b^ (SD)	Time to Run/Walk 1 Mile,^b^ Min (SD)
Underweight	173.14(a,b) (20.41)	4.93 (0.23)	0.75(a,b) (0.25)	0.68(b) (0.32)	11.25(a,b) (2.83)
Desirable weight	163.38(a) (33.22)	4.60 (0.43)	0.53(a) (0.26)	0.62(a) (0.23)	10.61(a) (2.64)
At risk for overweight	176.17(a,b) (27.17)	4.66 (0.33)	0.66(a) (0.26)	0.60(a) (0.24)	12.21(a,b) (2.60)
Overweight	188.57(b) (44.11)	4.65 (0.47)	0.84(b) (0.20)	0.82(b) (0.16)	12.96(a,b) (2.89)

BP indicates blood pressure; HbA1c, glycated hemoglobin.

a Underweight = less than the 5th percentile; desirable weight = 5th percentile to less than the 85th percentile; at risk for overweight = 85th percentile to less than the 95th percentile; overweight = 95th percentile or greater (defined in accordance with the recommendations of the Expert Committee Recommendations for Obesity Evaluation and Treatment) (19).

b Means with the same letters in parentheses are not significantly different.

**Table 6:** Logistic Regression, Predicting Hypertension Status Using Systolic, Diastolic, and Combined Blood Pressure Age-Specific Percentile Values in Two Inner-City Middle Schools, Los Angeles, California, 1999–2000 School Year

**Variable^a^**	**Hypertensive Systolic BP**		Hypertensive **Diastolic BP**		**Combined Hypertensive BP**	

	OR (95% CI)	*P* Value	OR (95% CI)	*P* Value	OR (95% CI)	*P* Value
African American	0.62 (0.27-1.45)	.27	2.56 (1.06-6.17)	.04	1.38 (0.64-2.94)	.41
Female	1.05 (0.46-2.40)	.91	0.70 (0.30-1.62)	.40	0.80 (0.38-1.70)	.56
12 Years of age	0.62 (0.24-1.58)	.32	1.36 (0.55-3.36)	.51	0.83 (0.36-1.91)	.66
Time to run/walk 1 mile, min	1.15 (0.99-1.33)	.06	0.92 (0.78-1.07)	.26	0.99 (0.86-1.13)	.86
Overweight	3.42 (1.50-7.80)	.004	8.01 (3.11-20.63)	< .001	7.04 (3.00-16.50)	< .001

BP indicates blood pressure; OR, odds ratio; CI, confidence interval.

a Referent for race/ethnicity was Latino; for sex, male; for weight status, desirable weight; for age, 11 years; for time to run/walk 1 mile, 7 minutes.
